# Validation of depressive symptoms, social support, and minority stress scales among gay, bisexual, and other men who have with men (GBMSM) in Nigeria, Africa: a mixed methods approach

**DOI:** 10.1186/s12889-020-09127-0

**Published:** 2020-06-29

**Authors:** Adedotun Ogunbajo, Stella Iwuagwu, Rashidi Williams, Katie B. Biello, Christopher W. Kahler, Theodorus G. M. Sandfort, Matthew J. Mimiaga

**Affiliations:** 1 Department of Behavioral and Social Sciences, Brown University School of Public Health, 121 South Main St., Box G-S121-3, Providence, RI 02912 USA; 2 Center for Health Equity, Brown School of Public Health, Providence, RI USA; 3 Centre for Right to Health, Abuja, Nigeria; 4 Equality Triangle for Health and Peoples Development Initiative, Warri, Delta Nigeria; 5 Department of Epidemiology, Brown University School of Public Health, Providence, RI USA; 6 The Fenway Institute, Fenway Health, Boston, MA USA; 7 HIV Center for Clinical and Behavioral Studies New York State Psychiatric Institute and Columbia University, New York, USA; 8 Department of Psychiatry and Human Behavior, Brown University Alpert Medical School, Providence, RI USA

**Keywords:** Minority stress, GBMSM, Nigeria, Mental health, Validity, Reliability, Gay and bisexual men, Social support, Depression, LGBT

## Abstract

**Background:**

Gay, bisexual, and other men who have sex with men (GBMSM) in Nigeria experience social marginalization, discrimination and violence due to their sexual identity, which may negatively impact physical, mental, and sexual health outcomes. Studies on GBMSM in Africa utilize measurement scales developed largely for populations in the Global North. The validity and reliability of these instruments—to our knowledge—have never been thoroughly investigated among GBMSM in Nigeria. The aim of the current study was to determine the validity and reliability of the English versions of the Center for Epidemiologic Studies Depression Scale (CESD-R), Multidimensional Scale of Perceived Social Support (MSPSS), and LGBT Minority Stress Measure among a large multi-state sample of GBMSM Nigeria.

**Methods:**

Between January and June 2019, we conducted cognitive interviews (*N* = 30) and quantitative assessments (*N* = 406) with GBMSM in Nigeria. The cognitive interviews assessed comprehension of scale items and elicited suggestions for scale modifications. The quantitative assessment was used to gather psychosocial health data and to evaluate psychometric properties and construct validity of the modified scales. We utilized confirmatory factor analysis to assess factor structure, correlation coefficients, and Cronbach’s alpha to examine scale validity and internal consistency.

**Results:**

Based on participant feedback from the cognitive interviews, we made slight modifications (i.e., culturally appropriate word substitutions) to all three scales. Results of quantitative analyses indicated good psychometric properties including high factor loadings, internal consistency and construct validity among the CESD-R, MSPSS, and LGBT Minority Stress Measure among GBMSM in Nigeria.

**Conclusion:**

These results suggests that modifying research scales to be more culturally relevant likely do not jeopardize their validity and reliability. We found that modified scales measuring depressive symptoms, perceived social support, and minority stress among GBMSM in Nigeria remained valid. More research is needed to explore whether the psychometric properties remain if the scales are translated into broken English (Pidgin) and other traditional Nigerian languages (Yoruba, Igbo and Hausa).

## Introduction

Nigerian gay, bisexual, and other men who have sex with men (GBMSM) experience marginalization, discrimination and violence due to their sexual orientation and same-sex sexual activity [1–3], which may negatively impact physical, mental, and sexual health outcomes [4, 5]. The minority stress model posits that the prejudice, discrimination, and stigma experienced by sexual and gender minority individuals—as a result of sexual or gender identity—contributes to higher levels of stress, which may lead to mental health problems [6] and sexual risk behaviors [7, 8]. Prior studies have found high levels of mental health problems (i.e. depression, anxiety, suicide ideation, and post-traumatic stress disorder (PTSD)) among Nigerian GBMSM [9, 10]. Consequently, it is important to explore these topics, especially considering the vulnerable and hostile situations Nigerian GBMSM are constantly confronted with.

According to the minority stress model, the pathway between sexual and gender minority stress and mental health problem may be moderated by social support and coping strategies [6]. Several studies have been conducted among GBMSM that provide evidence for the theoretical underpinnings and pathways asserted by this model [11–13]; additionally, similar findings have been observed among sexual minority women [13–15], and transgender populations [16–18]. Consequently, accurately measuring depressive symptoms, social support, and minority stress is important to appropriately intervening to improve the quality of life of Nigerian GBMSM.

Most quantitative studies conducted among African GBMSM utilize research instruments and scales developed and validated in the Global North (especially in North America and Europe) and include participants from those settings. Consequently, these scales may contain cultural references and colloquialism that may not be applicable, easily understood, or culturally relevant to African GBMSM. Formative research on the psychometric properties of these scales is essential to accurately quantify depressive symptoms, social support, and minority stress, and subsequently devise intervention strategies to effectively address these issues.

The aim of the current study was to adapt—after cognitive testing—and subsequently assess the validity, reliability, and psychometric properties of three widely used psychosocial measures in a large multi-state sample of GBMSM in Nigeria. The Center for Epidemiologic Studies Depression Scale (CESD-R) [19] is a 20-item scale used to screen for clinically significant depressive symptoms. The Multidimensional Scale of Perceived Social Support (MSPSS) [20], is a 12-item validated scale used to measure perceived social support from family, friends, and significant others. The LGBT Minority Stress Measure [21], is a 50-item scale developed to measure stress-related components of the minority stress model: prejudice events, victimization events, anticipation of rejection, identity concealment, internalized anti-LGBT stigma, everyday discrimination, and community support. These scales have been widely utilized to measure depressive symptoms, social support, and minority stress among GBMSM [22–25]. While many studies have investigated the psychometric characteristics of these scales, a vast majority have been conducted in the Global North (largely in the United States of America) [21, 26, 27]. The validity and reliability of these instruments—to our knowledge—have never been investigated among GBMSM in Nigeria.

## Methods

### Mixed-methods approach

We utilized a sequential exploratory mixed method design [28], which is a methodological approach that combines qualitative and quantitative data collection and analysis in phases. In the first phase, we collected qualitative data on the cultural relevancy of the unmodified research instruments (cognitive testing) and analyzed the data. Next, we modified the instruments, based on the feedback from participants, and carried out the quantitative phase, where we tested the psychometric properties of the modified research instruments.

### Cognitive testing

#### Participants and procedures

In January 2019, we recruited 30 GBMSM from Delta (*n* = 15) and Lagos (n = 15), Nigeria through local community-based organizations (CBOs) to participate in one-on-one cognitive interviews to assess cultural relevance and comprehension of the CESD-R, the MSPSS, and the LGBT Minority Stress Measure. Participants were asked to provide suggestions for modification of these scales to make them easily understandable by Nigerian GBMSM. Inclusion criteria for study participation were: 1) 18 years of age or older; 2) currently residing in Delta or Lagos; 3) cis-gender male; and 4) history of sex with another male. Peer educators at the two CBOs shared information about the cognitive testing with the target population at various community-centered events (e.g., HIV testing and counseling, health education, advocacy events, etc.) and provided study contact information to individuals who were interested. Study activities took place in private offices within our partner CBOs.

The theoretical groundings for our cognitive testing approach emanated from the question-answer model, which proposes that in order for participants to accurately answer a question, they must: 1) understand the question, 2) retrieve the necessary information from their long-term memory, 3) decide what information is necessary to respond to the question, and 4) answer the question [29]. First, we read out-loud the instructions for each scale to participants. Next, we read each item and probed whether participants understood what the question was asking. We had the participants repeat back what they believed the question was asking. Next, we asked how they would modify the question to be more easily understood by and relevant to GBMSM in Nigeria. This protocol was repeated for each item within a scale. Lastly, participants were asked what overall construct the scale aimed to measure. This protocol was repeated for each individual scale. All interviews were digitally recorded. Based on feedback from the cognitive interviews and iterative feedback from senior authors, the scales were modified and subsequently administered to a large, multi-state sample of GBMSM in Nigeria.

### Quantitative scale validation

#### Participants and procedures

Between March and June 2019, 406 GBMSM were recruited from Abuja (*n* = 107), Delta (*n* = 102), Lagos (*n* = 112), and Plateau (*n* = 85) through community-based organizations (CBOs) and snowball sampling. Peer educators, outreach workers, and key opinion leaders from CBOs based in the four study sites provided potential participants with information about the study and a study contact number. Individuals who showed interest in the study were screened for eligibility. Eligibility criteria were: 1) 18 years of age or older; 2) currently residing in one of four Nigerian states (Abuja, Delta, Lagos or Plateau); 3) identify as cis-gender male (i.e., participants who were assigned male sex at birth and currently identify as men); and 4) any self-reported history of sex (oral or anal) with another male. Eligible participants were asked to provide information about the study to other members of their social network. Data collection was conducted in the private offices of each CBO. Each participant provided verbal informed consent and completed the quantitative survey with the help of a trained research assistant. The survey took 1 to 1.5 h to complete. Upon completion of the survey, participants were compensated with 4000 Naira (equivalent to 10 US dollars). The study protocols were approved by the institutional review boards at Brown University and the Nigerian Institute of Medical Research.

#### Measures tested

##### Depressive symptoms

Depressive symptoms were assessed using the CESD-R scale [19], a 20-item self-report scale used to screen for clinically significant depressive symptoms. These instructions were given to participants prior to completing this scale: “Below is a list of the ways you might have felt or behaved. Please check the box to tell me how often you have felt this way in the past week.” The items were scored on a 4-point scale ranging from 0 “not at all or less than one day” to 3 “5-7 days or nearly every day for two weeks”, and summed, with a higher score indicating more severe depressive symptoms. We investigated the psychometric properties of the one-factor structure of the CESD-R to assess overall depressive symptoms, which has been demonstrated to have adequate data fit characteristics [26, 30].

##### Perceived social support

Perceived social support was assessed using the MSPSS [20], a 12-item self-report scale used to measure perceived social support from family, friends, and significant other. These instructions were given to participants prior to completing this scale: “We are interested in how you feel about the following statements. Read each statement carefully. Indicate how you feel about each statement.” The items were scored on a 7-point Likert scale ranging from 1 “very strongly disagree” to 7 “very strongly agree”. Scores were summed and higher scores indicated greater perceived social support. We investigated the psychometric properties of the three-factors structure of the MSPSS, which assesses three distinct sources of social support (family, friends, and significant other), which has been demonstrated to have adequate data fit characteristics [20, 27, 31].

##### Minority stress scales

Five distinct constructs within minority stress were assessed using the LGBT Minority Stress Measure [21]: community connectedness, internalized stigma, rejection anticipation, identity concealment, and victimization events. These instructions were given to participants prior to completing these scales: “The next few questions will ask you about the LGBT community. LGBT stands for Lesbian, Gay, Bisexual, and Transgender individuals. Please think about your own identity within the community and your relation with the LGBT community when answering these questions.” Community connectedness was assessed using five items and scored on a 5-point Likert scale ranging from 1 “Strongly Disagree” to 5 “Strongly Agree” with a higher score indicating higher levels of community connectedness. Internalized stigma was assessed using 3 items and scored on a 5-point Likert scale ranging from 1 "Strongly Disagree to 5 “Strongly Agree” with a higher score indicating higher levels of internalized stigma. Rejection anticipation was assessed using four items and scored on a 5-point Likert scale ranging from 1 “Never Happens” to 5 “Happens all the time” with a higher sore indicating higher levels of rejection anticipation. Identity concealment was assessed using four items and scored on a 5-point Likert scale ranging from 1 “Never Happens” to 5 “Happens all the time” with a higher sore indicating higher levels of identity concealment. Victimization events was assessed using three items and scored on a 5-point Likert scale ranging from 1 “Never Happens” to 5 “Happens all the time” with a higher sore indicating higher levels of self-reported experiences of victimization events. We investigated the psychometric properties of the five distinctive constructs of minority stress that we were interested in (community connectedness, internalized stigma, rejection anticipation, identity concealment, and victimization events).

##### Measures to assess construct validity

To assesses the validity of CESD-R, MSPSS, and the LGBT Minority Stress Measure, we chose two measures we hypothesized would be significantly (convergent validity) and non-significantly (divergent validity) correlated (Pearson correlation coefficient) with our measures.

**Convergent Validity**

The purpose of convergent validity is to assess whether the scales are significantly related as predicted [32]. The UCLA Loneliness Scale was used to assess convergent validity as past research has demonstrated significant positive associations between depressive symptoms, minority stress and loneliness [33, 34]; and a significant negative association between social support and loneliness [35]. Loneliness was assessed using the UCLA Loneliness Scale [36], an 8-item validated scale that measures various aspects of loneliness on a 4-point Likert scale ranging from 1 “Never” to 4 “Often”. Scores were summed and higher scores indicated greater perceived loneliness.

**Divergent Validity**

The purpose of divergent validity is to examine whether the construct of interest is different from a separate concept [32]. Healthcare utilization was used to assess divergent validity as we hypothesize that it would have smaller association with experiences of minority stress, depressive symptoms, and perceived social support. Healthcare utilization was assessed by asking participants: “When was the last time you went to a doctor for a medical check-up? A routine checkup is a general physical exam, not for a specific injury, illness or condition” with possible options response “within the last year,” “within the last two years,” “within the last five years,” “five years or more ago,” or “never”.

### Data analysis

#### Cognitive testing

All interviews were transcribed by a professional transcribing company based in Nigeria. As English is the official language of Nigeria, scales were administered in English and no translation services were necessary. We analyzed the cognitive interviews consistent with best practice recommendations [37, 38] and previous research [39, 40]. The transcripts were independently analyzed by one study team member. Analyses were structured around the constructs of the question-answer model explained above. Each question within each scale was analyzed independently. We complied a comprehensive list of all suggested changes for each individual question. Modifications were made to individual questions when two or more participants suggested changes to that question. Next, all suggested modifications were considered and a list of three or less possible revised questions were noted. After consultation with a group of experts—consisting of GBMSM and researchers who work with this population in Nigeria—the final version of the revised questions were reached. The most parsimonious and easily understandable questions were selected.

#### Confirmatory factor analysis

We used MPlus to conduct confirmatory factor analysis (CFA). CFA is a psychometric assessment that allows for testing of an a priori factor structure of a specific measurement instrument and estimation of latent constructs while correcting for measurement errors [32]. We conducted CFA, rather than exploratory factor analysis, because the scales we were validating have clearly defined subscales and constructs and have been widely utilized within the field of behavioral and public health research. Participants with any missing responses were excluded from the CFA. To assess fit of the model [41, 42], we examined the root mean square error of approximation (RSMEA) values (< 0.06 considered excellent and < 0.08 considered good); comparative fit index (CFI) and tucker-lewis index (TLI) values (< 0.95 considered excellent and < 0.90 considered good); and the akaike information criterion (AIC) assessed model parsimony, with a significant decrease in AIC suggesting a better fitting model.

## Results

### Cognitive interviews demographics (*N* = 30)

As seen in Table 1, participants ranged in age from 20 to 40 years (mean = 29.1, standard deviation [SD] = 5.3), and half (*n* = 15, 50.0%) of participants identified as gay/homosexual. Most participants (*n* = 19, 63.3%) reported their relationship status as single and more than three-fourths (*n* = 23, 76.7%) had a university education or higher.

**Table 1:** Sociodemographic characteristics of Cognitive Interview participants (*N* = 30)

Demographics	
	Mean (SD); N (%)
**Age (in years)**	29.1 (5.3)
**Current Sexual Orientation**	
Gay/Homosexual	15 (50.0%)
Bisexual	15 (50.0%)
**Current Relationship Status**	
Single, never been married	19 (63.3%)
Long-term relationship with a man	7 (23.3%)
Long-term relationship with a man	3 (10.0%)
Separated	1 (3.3%)
**Current Housing Status**	
Stable Housing	29 (96.7%)
Unstable Housing	1 (3.3%)
**Educational Attainment**	
Senior Secondary School (SSS) or lower	7 (23.3%)
Some University/Vocation Education	15 (50.0%)
University Degree	8 (26.7%)
**Current Employment Status**	
Employed	16 (53.3%)
Unemployed	14 (46.7%)

### Cognitive interview findings

Of the 20 statements contained in the CESD-R scale, 8 were modified (Table 2). A majority of the modifications constituted changing a few words to make the phrase more understandable (for example, we changed “I had trouble keeping my mind of what I was doing” to “I had trouble concentrating on what I was doing”). Only one of the statements was completely modified (“I could not get going” to “I lacked motivation”).

**Table 2:** Original and modified measurement scale items

Scale	Original Item	Modified Item
CESD-R	My appetite was poor	I didn’t have an appetite
	I could not shake off the blues	I could not change my bad mood (final item) I could not think straight (other suggested item) I could not think properly (other suggested item)
	I had trouble keeping my mind of what I was doing	I had trouble concentrating on what I was doing
	I could not get going	I lacked motivation
	I lost interest in my usual activities	I lost interest in my daily activities
	I felt fidgety	I felt nervous
	I wanted to hurt myself	I wanted to harm myself
	I had a lot of trouble getting to sleep	I had trouble sleeping
MSPSS	There is a special person who is around when I am in need	There is a significant other I can lean on
	There is a special person with whom I can share my joys and sorrows	There is a significant other who I can share my joys and sorrows with
	I get the emotional help and support I need from my family	I get the love and support I need from my family
	I have a special person who is a real source of comfort to me	I have a significant other who is a real source of comfort to me
	My friends really try to help me	My friends are there for me
	I can talk about my problems with my family	I can share my problems with my family
	I have friends with whom I can share my joys and sorrows	I have friends who I can share my joys and sorrows with
	There is a special person in my life who cares about my feelings	There is a significant other in my life who cares about my feelings
The LGBT Minority Stress Measure		
Community Connectedness	I feel like I am a part of the GBMSM community	I feel like I am a member of the LGBT community
	I feel that I could find information and pamphlets on GBMSM issues	I feel that I could find information, books, flyers on LGBT issues
	I feel that I could find professional services for GBMSM issues if I needed to	I feel that I could find friendly services for LGBT issues if I needed to
	I feel that I could find a public space that is supportive of GBMSM activities	I feel like there is a safe space where LGBT social activities can take place
Internalized Stigma	If I was offered the chance to be someone who is not GBMSM, I would accept the opportunity	If I could change from being LGBT to straight, I would
	I envy people who are not GBMSM.	I am jealous of people who are not LGBT
Rejection Anticipation	I brace myself to be treated disrespectfully because I am GBMSM	I prepare myself to be treated disrespectfully because I am LGBT
Identity Concealment	I avoid telling people about certain things in my life that might imply I am GBMSM	I avoid telling people about certain things in my life that might make them think I am LGBT
	I avoid talking about my romantic life because I do not want others to know I am GBMSM	I avoid talking about my love life because I do not want others to know I am LGBT
	I do not bring a date to social events because I do not want others to know I am GBMSM	I do not bring a date to social gathering/ parties because I do not want others to know I am LGBT
Victimization Events	I have been verbally harassed or called names because I am GBMSM	I have been called names or insulted because I am LGBT

Of the 12 statements contained in the MSPSS, 8 were modified (Table 2). In the significant other subscale, “special person” was replaced with “significant other”. The rest of the changes were minor word substitutions such as changing “I can talk about my problems with my family” to “I can share my problems with my family”.

Of the combined 16 statements contained in the 5 subscales of the LGBT Minority Stress Measure, 11 were modified (Table 2). A majority of the modifications constituted changing a few words to make the phrase more understandable (for example, we changed “I feel like I am a part of the LGBT community” to “I feel like I am a member of the LGBT community”). A few statements were completely changed (for example, we changed “If I was offered the chance to be someone who is not LGBT I would accept the opportunity” to “If I could change from being LGBT to be straight, I would.”

### Quantitative sample demographics (*N* = 406)

As seen in Table 3, participants ranged in age from 18 to 60 years (mean = 29.2, SD = 5.8), the majority (*n* = 238, 58.6%) identified as bisexual, and 61.6% were single. We had an ethnically diverse sample (20.3% were Igbo, 17.8% were Hausa, 17.7% were Yoruba, 15.7% were Urhobo, and many more ethnic groups were represented) Most (*n* = 238, 61.8%) participants reported experiencing high financial hardship and 22.3% reported a history of incarceration (*n* = 86). One-fourth (*n* = 99, 24.8%) of participants reported living with HIV and one third (*n* = 124, 32.3%) reported a sexually transmitted infection diagnosis in the previous year.

**Table 3:** Sociodemographic characteristics of Quantitative Assessment participants (*N* = 406)

Demographics	
	Mean (SD); N (%)
**Age (in years)**	29.2 (5.8)
**Current Sexual Orientation**	
Gay/Homosexual	160 (39.4%)
Bisexual	238 (58.6%)
**Current Relationship Status**	
Single	250 (61.6%)
Not Single	150 (36.9%)
**Religious Affiliation**	
Christian	253 (62.3%)
Muslim	116 (28.6%)
Other	30 (7.4%)
**Monthly income (in Naira)**	
0–10,000	105 (25.9%)
10,000-30,000	106 (26.1%)
30,000-50,000	81 (20.0%)
50,000-100,000	54 (13.3%)
100,000+	49 (12.1%)
**Employment Status**	
Employed	319 (78.6%)
Unemployed	81 (20.0%)

### Confirmatory factor analysis results (*N* = 406)

#### CESD-R

All items significantly loaded onto the one-factor depression construct except item #9 (I slept much more than usual), (β = 0.25) (Table 4). The fit indices for the one-factor model were acceptable (RMSEA = 0.10; CFI = 0.82; TLI = 0.80). This provides evidence that the CESD-R is a reasonable instrument to ascertain depressive symptoms among Nigerian GBMSM.

**Table 4:** Standardized factor loadings from confirmatory factor analysis for the Center for Epidemiologic Studies Depression Scale (CESD-R)

Item	1 Factor
	Depression **(α = 0.93)**
1. I could not change my bad mood^a^	0.665
2. I felt depressed	0.710
3. I felt sad	0.656
4. Nothing made me happy	0.709
5. I lost interest in my daily activities^a^	0.710
6. I didn’t have an appetite^a^	0.452
7. I lost a lot of weight without trying to	0.617
8. My sleep was restless	0.679
9. I slept much more than usual	**0.246**
10. I had trouble sleeping	0.646
11. I had trouble concentrating on what I was doing	0.680
12. I could not focus on the important things	0.762
13. I felt like a bad person	0.668
14. I did not like myself	0.651
15. I lacked motivation^a^	0.649
16. I was tired all the time	0.684
17. I felt like I was moving too slowly	0.659
18. I felt nervous^a^	0.743
19. I wished I were dead	0.595
20. I wanted to harm myself^a^	0.466

^a^modified version of original question

#### MSPSS

All items significantly loaded onto their respective factors (Table 5). The three-factor model measures three distinct sources of perceived social support (family, friends, and significant other). The fit indices for the three-factor model were acceptable (RMSEA = 0.09; CFI = 0.92; TLI = 0.90). The good fit statistics and multidimensional nature of social support leads us to conclude that the three-factor model is parsimonious.

**Table 5:** Standardized factor loadings from confirmatory factor analysis for Multidimensional Scale of Perceived Social Support (MSPSS)

Item	3 Factor		
	Significant Other (α = 0.81)	Family (α = 0.80)	Friends (α = 0.82)
1. There is a significant other I can lean on^a^	0.672		
2. There is a significant other who I can share my joys and sorrows with^a^	0.675		
3. I have a significant other who is a real source of comfort to me^a^	0.790		
4. There is a significant other in my life who cares about my feelings^a^	0.708		
5. My family really tries to help me		0.820	
6. I get the love and support I need from my family^a^		0.835	
7. I can share my problems with my family^a^		0.581	
8. My family is willing to help me make decisions		0.579	
9. My friends are there for me^a^			0.755
10. I can count on my friends when things go wrong			0.821
11. I have friends who I can share my joys and sorrows with^a^			0.674
12. I can talk about my problems with my friends			0.673

^a^modified from original question

#### LGBT minority stress scales

All items significantly loaded onto their respective factors (Table 6). The five-factor model measures five distinct experiences of minority stress (community connectedness, internalized stigma, rejection anticipation, identity concealment, and victimization events). The good fit statistics (RMSEA = 0.08; CFI = 0.91; TLI = 0.90) and multidimensional nature of minority stress leads us to conclude that these measures accurately assessed various dimensions of experiences of minority stress among Nigerian GBMSM.

**Table 6:** Standardized factor loadings from confirmatory factor analysis for the LGBT Minority Stress Measure

Item	5 Factor				
	Community Connectedness (α = 0.86)	Internalized Stigma (α = 0.80)	Rejection Anticipation (α = 0.72)	Identity Concealment (α = 0.86)	Victimization Events (α = 0.92)
1. I feel connected to other LGBT people	0.697				
2. I feel like I am a member of the LGBT community^a^	0.673				
3. I feel that I could find information, books, flyers on LGBT issues^a^	0.849				
4. I feel that I could find friendly services for LGBT issues if I needed to^a^	0.801				
5. I feel like there is a safe space where LGBT social activities can take place^a^	0.664				
6. If I could change from being LGBT to be straight, I would^a^		0.841			
7. I wish I wasn’t LGBT		0.904			
8. I am jealous of people who are not LGBT^a^		0.554			
9. When I meet someone new, I worry that they secretly do not like me because I am LGBT			0.645		
10. I prepare myself to be treated disrespectfully because I am LGBT^a^			0.550		
11. I expect that others will not accept me because I am LGBT			0.764		
12. I worry about what will happen if people find out I am LGBT			0.593		
13. I avoid telling people about certain things in my life that might make them think I am LGBT^a^				0.865	
14. I avoid talking about my love life because I do not want others to know I am LGBT^a^				0.888	
15. I do not bring a date to social gathering/ parties because I do not want others to know I am LGBT^a^				0.693	
16. I limit what I share on social media, or who can see it, because I do not want others to know I am LGBT				0.591	
17. I have been called names or insulted because I am LGBT^a^					0.864
18. Others have threatened to harm me because I am LGBT					0.905
19. I have been bullied by others because I am LGBT					0.898

^a^modified from original question

### Scale properties (*N* = 406)

Scores on the CESD-R (20 items) ranged from 0 to 55 (M = 11.4, SD = 12.2). Internal consistency was high (Cronbach’s α = 0.93). Scores on the MSPSS (12 items) ranged from 12 to 84 (M = 58.4, SD = 12.6). Internal consistency was high (Cronbach’s α = 0.86). Scores on the community connectedness subscale (5 items) ranged from 5 to 25 (M = 19.8, SD = 4.5). Internal consistency was high (Cronbach’s α = 0.86). Scores on the internalized stigma subscale (3 items) ranged from 3 to 15 (M = 8.0, SD = 3.5). Internal consistency was high (Cronbach’s α = 0.80). Scores on the rejection anticipation subscale (4 items) ranged from 4 to 20 (M = 9.8, SD = 4.0). Internal consistency was acceptable (Cronbach’s α = 0.72). Scores on the identity concealment subscale (4 items) ranged from 4 to 20 (M = 13.1, SD = 4.8). Internal consistency was high (Cronbach’s α = 0.86). Scores on the victimization events subscale (3 items) ranged from 3 to 15 (M = 5.4, SD = 3.2). Internal consistency was very high (Cronbach’s α = 0.92).

#### Construct validity analysis

To evaluate the convergent validity (Table 7), correlations (Pearson’s coefficients) were conducted between the CESD-R, the MSPSS, the LGBT Minority Stress Measure, and the UCLA Loneliness Scale. We hypothesized that there will be a positive significant relationship between depressive symptoms, minority stress, and loneliness. We also hypothesized a significant inverse relationship between perceived social support and loneliness. Upon calculation of Pearson’s coefficient, the UCLA Loneliness Scale was found to be correlated, but not strongly, in the expected direction with CESD-R (*r* = 0.38, *p* < 0.01), perceived social support (family [*r* = − 0.23, *p* < .01], friends [*r* = − 0.26, *p* < 0.01], and significant other [*r* = − 0.20, *p* < 0.01]) and all but one of the minority stress scales (community connectedness [*r* = − 0.09, not significant], internalized stigma [*r* = 0.10, *p* < 0.05], rejection anticipation [*r* = 0.23, *p* < 0.01], identity concealment [*r* = 0.14, *p* < 0.01], and victimization events [*r* = 0.19, *p* < 0.01]), thereby demonstrating evidence for convergent validity. Additionally, the social support and minority stress subscales were highly correlated with each other (|r| = 0.23–0.48), *p* < 0.01), providing evidence for concurrent validity.

**Table 7:** Correlation demonstrating convergent and divergent validity between scales and validity measures

Measure	1	2	3	4	5	6	7	8	9	10	11
1. CESD-R	1	−.26**	−.04	.15**	.37**	.17**	.24**	.23**	.27**	0.38**	0.02
2. MSPSS (family)		1	.48**	0.53**	.23**	−0.18**	−0.17**	−0.48**	−0.28**	0.23**	−0.01
3. MSPSS (friends)			1	0.56**	.25**	−0.20**	−0.23**	−0.17**	−0.29**	0.26**	−0.07
4. MSPSS (significant other)				1	0.19**	0.10	0.14	− 0.19**	− 0.24**	0.20**	− 0.05
5. Community Connectedness					1	−0.29**	−0.27**	− 0.25**	− 0.38**	0.09	0.09
6. Internalized Stigma						1	0.29**	0.25**	0.33**	0.10*	−0.03
7. Rejection Anticipation							1	0.27**	0.24**	0.23**	0.06
8. Identity Concealment								1	0.30**	0.14*	−0.02
9. Victimization Events									1	0.19**	0.05
10. Loneliness										1	0.04
11. Healthcare Utilization											1

*P < .05, **P < .01

To evaluate discriminant validity (Table 7), correlations (Pearson’s coefficients) were conducted between CESD-R, MSPSS, LGBT Minority Stress Measure, and healthcare utilization. We hypothesized that there will be no statistically significant relationship between depressive symptoms, perceived social support, minority stress and healthcare utilization. Upon calculation of Pearson’s coefficient, healthcare utilization was found to be not strongly correlated with the CESD-R (*r* = 0.02, not significant), perceived social support (family [*r* = − 0.01, not significant], friends [*r* = − 0.07, not significant], and significant other [*r* = − 0.05, not significant]) and all the minority stress scales (community connectedness [*r* = 0.09, not significant], internalized stigma [*r* = − 0.03, not significant], rejection anticipation [*r* = 0.06, not significant], identity concealment [*r* = − 0.02, not significant], and victimization events [*r* = 0.05, not significant]), thereby demonstrating strong evidence for discriminant validity.

## Discussion

This is the first study, as far as we are aware, to investigate the psychometric properties of key psychosocial research instruments among Nigerian GBMSM. Confirmatory factor analysis, internal consistency, and construct validity all suggest that the CESD-R, the MSPSS, and the LGBT Minority Stress Measure have strong validity and reliability in this sample, even after the modifications (21 out of 48 total question items were modified). These findings are especially strong given the geographical and ethnic group diversity represented in our sample. Results suggest that modified versions of psychosocial scales can accurately measure the same constructs as the original scales even after being modified to be more culturally relevant. The structural validity of these scales has major implications for use in future behavioral research and intervention studies among Nigerian and generally among African GBMSM.

We found that the CESD-R had high factor loadings, internal consistency and construct validity. However, an item related to sleep quality (‘I slept much more than usual’) had poor factor loading on both the overall depression scale and sleep construct within the overall scale. This might be attributable to a differing cultural conceptualization of sleep, where quality of sleep may vary vastly on basis of age, geographical location, ethnic group membership, amongst other factors. It is important to understand that sleep disturbance, as a result of depressive symptoms, can manifest as either hypersomnia or insomnia. The sleep-related question in the CESD-R only assesses hypersomnia, and not insomnia, which may partially explain the observed low factor loading. We found the one-factor measurement of depressive symptoms to be parsimonious, providing more evidence that the CESD-R might be a reliable scale to measure depressive symptoms among Nigerian GBMSM. This is especially relevant since previous studies have found high prevalence of depressive symptoms among Nigerian GBMSM [9, 10].

Similar to the CESD-R, the MSPSS had sound psychometric properties, which suggests its’ potential to accurately measure perceived social support from three distinct sources—family, friends, and significant other—among Nigerian GBMSM. This is of particular importance as social support has been hypothesized as a potential moderator of the association between experiences of minority stress and mental health problems in sexual minority communities [17, 43]. Perceived social support might reduce or diminish the effects of minority stress on mental health problems among individuals with high levels of perceived social support compared to individuals with low levels of perceived social support. Consequently, effectively measuring levels of perceived social support might help aid the design of interventions to help Nigerian GBMSM buffer the stress associated with their sexual orientation by identifying possible sources of social support and coping mechanisms.

We found that the LGBT Minority Stress Measure provided an accurate measurement of the various aspects of minority stress (community connectedness, internalized stigma, rejection anticipation, identity concealment, and victimization events). There was further evidence that each subscale independently measured a specific construct of minority stress. This finding enables researchers who are interested in specific constructs within minority stress to administer that specific subscale independent of the longer, comprehensive scale.

Our findings should be interpreted in the context of some limitations. While the sample was geographically and demographically diverse, the scales were evaluated among a sample mainly recruited through GBMSM community-based organizations and GBMSM social networks. This sampling frame limits our ability to generalize our findings to GBMSM who do not seek services at these organizations or who are outside of the social networks sampled. Further, social desirability bias may have influenced participants’ responses because the assessment was completed together with trained research assistants. Additionally, while we conducted cognitive interviews prior to administering the amended scales, we did not assess test-retest reliability, which would have provided stronger evidence of the validity of the scales after modification.

Future studies should investigate whether these psychometric properties hold for scales that have been translated into Nigerian pidgin English or native languages (Yoruba, Igbo, & Hausa). Translating these scales into the major local languages will broaden the reach of public health research by allowing individuals who feel comfortable communicating in these local languages to participate. It is also important to investigate the temporality of minority stress and its’ effects on depressive symptoms, and social support, which is best accomplished by conducting longitudinal studies.

## Conclusion

The current study provides further evidence that cultural adaptation of research instruments does not jeopardize the validity and reliability of the original scales. If the goal of public health research is to prevent disease on a population level, it is incumbent upon population health researchers to ensure that the measurement scales that are being utilized are culturally relevant and have sound psychometric properties. Our study provides evidence that both goals can be successfully accomplished.

## Data Availability

Data cannot be shared publicly because of the sensitive nature of the research subjects and the context in which the research was conducted. However, a codebook of data analysis procedures which allows replication of the reported results has been deposited for public access at the Brown University depository and is available here: 10.26300/bmq7-9k58.

## Abbreviations

GBMSM: Gay, bisexual, and other men who have sex with men
PTSD: Post-traumatic stress disorder
CESD-R: Center for Epidemiologic Studies Depression Scale
MSPSS: Multidimensional Scale of Perceived Social Support
CBOs: Community-based organizations
CFA: Confirmatory factor analysis

## References

[1] Sekoni AO, Ayoola OO, Somefun EO (2015). Experiences of social oppression among men who have sex with men in a cosmopolitan city in Nigeria. HIV/AIDS (Auckland, NZ).

[2] Allman D, Adebajo S, Myers T, Odumuye O, Ogunsola S (2007). Challenges for the sexual health and social acceptance of men who have sex with men in Nigeria. Cult Health Sex.

[3] Melhado L (2015). In Nigeria, anti-gay law associated with increased stigma and discrimination. Int Perspect Sex Reprod Health.

[4] Makanjuola O, Folayan MO, Oginni OA (2018). On being gay in Nigeria: discrimination, mental health distress, and coping. J Gay Lesbian Mental Health.

[5] Oginni OA, Mosaku KS, Mapayi BM, Akinsulore A, Afolabi TO (2018). Depression and associated factors among gay and heterosexual male university students in Nigeria. Arch Sex Behav.

[6] Meyer IH, Frost D (2013). Minority stress and the health of sexual minorities. Handbook of psychology and sexual orientation.

[7] Fields EL, Bogart LM, Galvan FH, Wagner GJ, Klein DJ, Schuster MA (2013). Association of discrimination-related trauma with sexual risk among HIV-positive African American men who have sex with men. Am J Public Health.

[8] Hidalgo MA, Cotten C, Johnson AK, Kuhns LM (2013). Garofalo R: ‘yes, I am more than just that’: gay/bisexual young men residing in the United States discuss the influence of minority stress on their sexual risk behavior prior to HIV infection. Int J Sex Health.

[9] Ogunbajo A, Oke T, Jin H, Rashidi W, Iwuagwu S, Harper GW, Biello KB, Mimiaga MJ (2019). A syndemic of psychosocial health problems is associated with increased HIV sexual risk among Nigerian gay, bisexual, and other men who have sex with men (GBMSM). AIDS Care.

[10] Rodriguez-Hart C, Bradley C, German D, Musci R, Orazulike I, Baral S, Liu H, Crowell TA, Charurat M, Nowak RG (2018). The synergistic impact of sexual stigma and psychosocial well-being on HIV testing: a mixed-methods study among Nigerian men who have sex with men. AIDS Behav.

[11] Meyer IH (1995). Minority stress and mental health in gay men. J Health Soc Behav.

[12] Hatzenbuehler ML, Nolen-Hoeksema S, Erickson SJ (2008). Minority stress predictors of HIV risk behavior, substance use, and depressive symptoms: results from a prospective study of bereaved gay men. Health Psychol.

[13] Feinstein BA, Goldfried MR, Davila J (2012). The relationship between experiences of discrimination and mental health among lesbians and gay men: an examination of internalized homonegativity and rejection sensitivity as potential mechanisms. J Consult Clin Psychol.

[14] Lehavot K, Simoni JM (2011). The impact of minority stress on mental health and substance use among sexual minority women. J Consult Clin Psychol.

[15] Kuyper L, Fokkema T (2010). Loneliness among older lesbian, gay, and bisexual adults: the role of minority stress. Arch Sex Behav.

[16] Testa RJ, Michaels MS, Bliss W, Rogers ML, Balsam KF, Joiner T (2017). Suicidal ideation in transgender people: gender minority stress and interpersonal theory factors. J Abnorm Psychol.

[17] Scandurra C, Amodeo AL, Valerio P, Bochicchio V, Frost DM (2017). Minority stress, resilience, and mental health: a study of Italian transgender people. J Soc Issues.

[18] Rood BA, Reisner SL, Surace FI, Puckett JA, Maroney MR, Pantalone DW (2016). Expecting rejection: understanding the minority stress experiences of transgender and gender-nonconforming individuals. Transgender Health.

[19] Eaton WW, Smith C, Ybarra M, Muntaner C, Tien A (2004). Center for Epidemiologic Studies Depression Scale: review and revision (CESD and CESD-R).

[20] Zimet GD, Dahlem NW, Zimet SG, Farley GK (1988). The multidimensional scale of perceived social support. J Pers Assess.

[21] Outland PL. Developing the LGBT minority stress measure: Colorado State University. Libraries; 2016.

[22] Hussen SA, Easley KA, Smith JC, Shenvi N, Harper GW, Camacho-Gonzalez AF, Stephenson R, del Rio C (2018). Social capital, depressive symptoms, and HIV viral suppression among young black, gay, bisexual and other men who have sex with men living with HIV. AIDS Behav.

[23] Mimiaga MJ, Reisner SL, Cranston K, Isenberg D, Bright D, Daffin G, Bland S, Driscoll MA, VanDerwarker R, Vega B (2009). Sexual mixing patterns and partner characteristics of black MSM in Massachusetts at increased risk for HIV infection and transmission. J Urban Health.

[24] Ogunbajo A, Iwuagwu S, Williams R, Biello K, Mimiaga MJ (2019). Awareness, willingness to use, and history of HIV PrEP use among gay, bisexual, and other men who have sex with men in Nigeria. PLoS One.

[25] Fisher CM, Woodford MR, Gartner RE, Sterzing PR, Victor BG (2019). Advancing research on LGBTQ microaggressions: a psychometric scoping review of measures. J Homosex.

[26] Van Dam NT, Earleywine M (2011). Validation of the Center for Epidemiologic Studies Depression Scale—Revised (CESD-R): pragmatic depression assessment in the general population. Psychiatry Res.

[27] Canty-Mitchell J, Zimet GD (2000). Psychometric properties of the multidimensional scale of perceived social support in urban adolescents. Am J Community Psychol.

[28] Creswell JW, Clark VLP (2017). Designing and conducting mixed methods research: sage publications.

[29] Tourangeau R (1984). Cognitive sciences and survey methods. Cogn Aspects Surv Methodol.

[30] Edwards MC, Cheavens JS, Heiy JE, Cukrowicz KC (2010). A reexamination of the factor structure of the Center for Epidemiologic Studies Depression Scale: is a one-factor model plausible?. Psychol Assess.

[31] Kazarian SS, McCabe SB (1991). Dimensions of social support in the MSPSS: factorial structure, reliability, and theoretical implications. J Community Psychol.

[32] Boateng GO, Neilands TB, Frongillo EA, Melgar-Quiñonez HR, Young SL (2018). Best practices for developing and validating scales for health, social, and behavioral research: a primer. Front Public Health.

[33] Su X, Zhou AN, Li J (2018). Shi L-e, Huan X, Yan H, Wei C: depression, loneliness, and sexual risk-taking among HIV-negative/unknown men who have sex with men in China. Arch Sex Behav.

[34] Grov C, Golub SA, Parsons JT, Brennan M, Karpiak SE (2010). Loneliness and HIV-related stigma explain depression among older HIV-positive adults. AIDS Care.

[35] Kao JC, Chuong A, Reddy MK, Gobin RL, Zlotnick C, Johnson JE (2014). Associations between past trauma, current social support, and loneliness in incarcerated populations. Health Justice.

[36] Hays RD, DiMatteo MR (1987). A short-form measure of loneliness. J Pers Assess.

[37] Beatty PC, Willis GB (2007). Research synthesis: the practice of cognitive interviewing. Public Opin Q.

[38] Fowler FJ, Lloyd SJ, Cosenza CA, Wilson IB (2016). Coding cognitive interviews: an approach to enhancing the value of cognitive testing for survey question evaluation. Field Methods.

[39] Reisner SL, Conron KJ, Tardiff LA, Jarvi S, Gordon AR, Austin SB (2014). Monitoring the health of transgender and other gender minority populations: validity of natal sex and gender identity survey items in a US national cohort of young adults. BMC Public Health.

[40] Wilson IB, Fowler FJ, Cosenza CA, Michaud J, Bentkover J, Rana A, Kogelman L, Rogers WH (2014). Cognitive and field testing of a new set of medication adherence self-report items for HIV care. AIDS Behav.

[41] Marsh HW, Hau K-T, Wen Z (2004). In search of golden rules: comment on hypothesis-testing approaches to setting cutoff values for fit indexes and dangers in overgeneralizing Hu and Bentler's (1999) findings. Struct Equ Model.

[42] Brown TA (2014). Confirmatory factor analysis for applied research: Guilford publications.

[43] Wong CF, Schrager SM, Holloway IW, Meyer IH, Kipke MD (2014). Minority stress experiences and psychological well-being: the impact of support from and connection to social networks within the Los Angeles house and ball communities. Prev Sci.

